# webSDA: a web server to simulate macromolecular diffusional association

**DOI:** 10.1093/nar/gkv335

**Published:** 2015-04-16

**Authors:** Xiaofeng Yu, Michael Martinez, Annika L. Gable, Jonathan C. Fuller, Neil J. Bruce, Stefan Richter, Rebecca C. Wade

**Affiliations:** 1 Molecular and Cellular Modeling group, Heidelberg Institute for Theoretical Studies (HITS), Heidelberg, Baden-Württemberg, Germany; 2 Center for Molecular Biology (ZMBH), DKFZ-ZMBH Alliance, Heidelberg University, Heidelberg, Baden-Württemberg, Germany; 3 Interdisciplinary Center for Scientific Computing (IWR), Heidelberg University, Heidelberg, Baden-Württemberg, Germany

## Abstract

Macromolecular interactions play a crucial role in biological systems. Simulation of diffusional association (SDA) is a software for carrying out Brownian dynamics simulations that can be used to study the interactions between two or more biological macromolecules. webSDA allows users to run Brownian dynamics simulations with SDA to study bimolecular association and encounter complex formation, to compute association rate constants, and to investigate macromolecular crowding using atomically detailed macromolecular structures. webSDA facilitates and automates the use of the SDA software, and offers user-friendly visualization of results. webSDA currently has three modules: ‘SDA docking’ to generate structures of the diffusional encounter complexes of two macromolecules, ‘SDA association’ to calculate bimolecular diffusional association rate constants, and ‘SDA multiple molecules’ to simulate the diffusive motion of hundreds of macromolecules. webSDA is freely available to all users and there is no login requirement. webSDA is available at http://mcm.h-its.org/webSDA/.

## INTRODUCTION

Macromolecular interactions, such as protein–protein interactions and protein–DNA interactions, are key to the function of biological systems. Studying macromolecular diffusional association computationally can give insights into biological interactions. Simulation of diffusional association (SDA) is a Brownian dynamics simulation software which can be used to perform macromolecular docking, to compute bimolecular association rate constants, and to simulate molecular diffusion for systems of many macromolecules (1–3). Intermolecular forces are computed from atomic-detail molecular structures. SDA has been used to generate diffusional encounter complexes for a wide range of macromolecular complexes, including those of barnase and barstar (2,4), cytochrome P450 enzymes and their redox protein partners (5–7), methionine aminopeptidase and the ribosome (8), and the linker histone H5 and the nucleosome (9). Moreover, SDA has been used to calculate association rate constants (1,2,10,11) and simulate the diffusive motion of hundreds of macromolecules in solution (3,12–15). All these functions of SDA have been extensively validated against experimental data. Although SDA is made available for standalone use, it cannot be used easily by non-experts because of its multitude of different parameters and options, many of which are interdependent. Setting these parameters correctly and understanding their function demands a good understanding of Brownian dynamics simulations and the structure of the SDA software.

webSDA is a web server which aims to eliminate these obstacles and make SDA's functionalities available to a broader audience. webSDA supports the generation of diffusional encounter complexes (‘SDA docking’), the calculation of biomolecular diffusional association rate constants (‘SDA association’) and the simulation of the diffusive motion of several hundred macromolecules (‘SDA multiple molecules’). The flexibility of the solutes can be accounted for by providing several different conformations for one of the solutes (‘SDA docking’ and ‘SDA association’) or each of the solutes (‘SDA multiple molecules’). It allows users to set up and run SDA simulations. webSDA automates the preparation of input files by generating input parameters automatically. If one parameter is used in multiple input files, it only needs to be defined once. Input files generated by webSDA can also be downloaded for use with the standalone version of SDA.

## webSDA WORKFLOW

**Figure 1.**
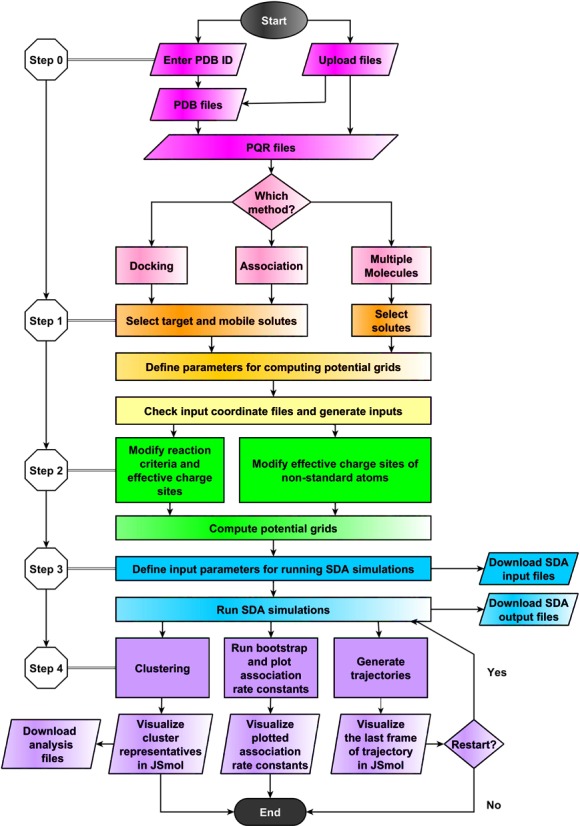
Workflow of webSDA.

The workflow of webSDA is shown in Figure 1.

### Step 0

To begin a simulation, users may enter the PDB identifiers of the macromolecules that they wish to simulate or upload coordinate files for the macromolecules in PDB or PQR format. PQR format files contain the atomic coordinates, formatted as in PDB files from the Protein Data Bank (www.rcsb.org/pdb), with partial atomic charges (*Q*) and atomic radii (*R*) instead of *B*-factors and occupancies. If PDB files are used, PQR files will be generated using the PDB2PQR software, version 2.0.0 (16), with the AMBER force field and default parameters. Users can also generate their own PQR files using the PDB2PQR web server (16). After uploading coordinate files, users select one of the SDA methods: ‘SDA docking’, ‘SDA association’ or ‘SDA multiple molecules’. Users then follow a sequence of steps to set up and run an SDA job, guided by hints on the webSDA website. As an example, the following steps show how to run an ‘SDA docking’ simulation.

### Step 1

Select target and mobile solutes: users need to choose a target solute and a mobile solute for the ‘SDA docking’ job.

### Step 2

Modify reaction criteria and effective charge sites: reaction criteria are distances between defined points in the solute molecules that are required to be satisfied for an encounter complex to be recorded in an SDA simulation. The default reaction criterion for ‘SDA docking’ is predefined for the centers of geometry of the solutes and requires that they approach within a distance equal to the sum of the maximum extensions of the two solutes plus 12 Å. The reaction criteria can be changed in the web interface and can be used to impose additional distance constraints which can be derived, e.g. from bioinformatics analysis or experimental data, such as FRET and mutagenesis. Effective charges are used in combination with electrostatic potential grids to approximate the electrostatic interactions between molecules (17). Users can modify the effective charge sites for non-standard atoms, such as found in low-molecular weight compounds, in the web interface.

### Step 3

Define input parameters for running SDA simulations: the automatically generated SDA parameters are given to users. Users can modify the SDA input parameters and run SDA simulations on the server. webSDA checks the input parameters that users have changed and helps users to set correct parameters. They may upload their own files (SDA input file, reaction criteria file and effective charge sites file) to run their SDA simulations on the web server. The web server can be used to run short jobs. The SDA input file can also be generated and downloaded together with the other files to run with the standalone version of the SDA software, which is also freely available.

### Step 4

Clustering: for ‘SDA docking’, the recorded diffusional encounter complexes are clustered and the cluster representatives can be visualized with JSmol on the web server or downloaded as described below.

## OUTPUT

**Figure 2.**
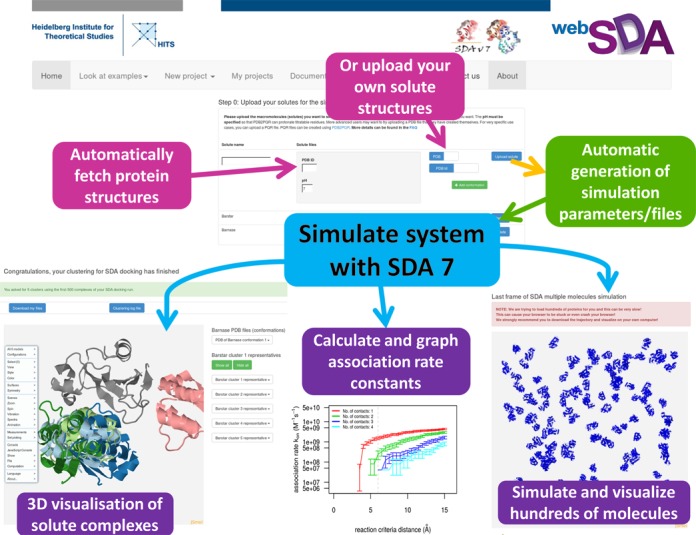
Screenshots of webSDA showing key features of webSDA.

Besides the option to download output files, webSDA offers a convenient visualization of the results. An example is given in Figure 2. For the results of ‘SDA docking’, JSmol is used for visualization of the cluster representatives of the diffusional encounter complexes. This provides users with an easy way to view encounter complexes and decide which complexes to download for further analyses. For ‘SDA association’, the calculated association rate constants are plotted using the R language, enabling users to visualize calculated association rate constants immediately. For ‘SDA multiple molecules’, the JSmol plug-in is employed to enable users to visualize the final snapshot of their simulations.

webSDA assigns each user a universally unique identifier (UUID). This enables users to revisit all projects that they have run in the ‘My projects’ page. There is a link associated with this page and users can send this link to colleagues to share projects.

## EXAMPLES OF webSDA USAGE

webSDA gives results consistent with the standalone version of SDA, which has been extensively validated against experimental data (1,8,12). webSDA provides example cases for each method. In Figure 2, we illustrate the performance of webSDA for the simulation of the association of barnase and barstar (PDB ID: 1BRS) and the simulation of many barnase molecules. With the ‘SDA docking’ method, cluster representatives of the docked barnase-barstar complexes in good agreement with the crystal structure of the complex and results obtained by Motiejunas *et al*. (4) are obtained.

It should be kept in mind that ‘SDA docking’ uses Brownian dynamics simulations to generate diffusional encounter complexes rather than fully docked complexes. The encounter complexes can be refined by molecular dynamics simulation to generate fully bound complexes, as shown in (4), but currently webSDA does not support running molecular dynamics simulation.

The ‘SDA association’ function is another important function of webSDA. For the barnase and barstar complex at an ionic strength of 50 mM and pH 7, the association rate constant computed with ‘SDA association’ is 4.1 × 10^8^ M^−1^s^−1^ (averaged from three webSDA runs: 4.0 × 10^8^, 4.0 × 10^8^ and 4.3 × 10^8^ M^−1^s^−1^), which is comparable with experimental data (3 × 10^8^ M^−1^s^−1^) (18) (Figure 2) and the simulation results reported by Gabdoulline and Wade (1).

The ‘SDA multiple molecules’ function of webSDA can be used to perform Brownian dynamics simulations of many molecules. This function of SDA has been used to study the behaviour of proteins in crowded environments and is shown to be able to reproduce experimental data (3,12–15). The long-time self-diffusion coefficients, second virial coefficients and structure factors, calculated by ‘SDA multiple molecules’, agree with experimental values.

There are several web servers (19–24) providing similar functionality to ‘SDA docking’ but they are often limited to proteins with standard amino acid residues. ‘SDA docking’ supports protein, DNA and RNA, and small molecules, such as a cofactor, can be treated as part of a macromolecular structure. For the calculation of association rate constants, the TransComp server (http://pipe.sc.fsu.edu/transcomp/) (25) computes association rate constants for protein–protein and protein–RNA complexes. ‘SDA multiple molecules’ is, to the best of our knowledge, the first web server for performing simulations of hundreds to thousands of macromolecules, which can be used to study concentrated protein solutions, and to investigate macromolecular crowding, or other phenomena involving large numbers of macromolecules.

## TECHNICAL OVERVIEW

The webSDA interface uses Javascript and HTML5 to control user interface (UI) elements. The server uses the Play framework version 2 and Apache commons.exec to manage user sessions and server resources. PDB2PQR version 2.0.0 is used to convert PDB files to PQR files (16,26). BioJava is used to process PDB ID and download PDB files. Python scripts (Biopython) are used to check PQR files and generate input parameters. SDA 7 (http://mcm.h-its.org/sda7/) is used to process PQR files, to generate grids (along with the Adaptive Poisson–Boltzmann Software (27) to compute electrostatic potential grids), and to perform simulations.

Currently, the maximum run-time of a job on the webserver is 24 h and the output files for SDA runs that execute for this time are written and provided to users. A queuing system using the Terascale Open-source Resource and QUEue Manager (TORQUE) is used to manage the submission of webSDA jobs to a backend compute cluster.

## DISCUSSION AND OUTLOOK

webSDA is a web server to perform Brownian dynamics simulations of biomacromolecules. ‘SDA docking’ allows users to generate diffusional encounter complexes of two solutes (proteins, DNA or RNA). ‘SDA association’ can be used to compute the bimolecular association rate constant for a specified complex and can be combined with ‘SDA docking’ when the structure of the complex is unknown. ‘SDA multiple molecules’ is helpful for studying the diffusive behaviour of hundreds of molecules.

webSDA automates the widely used SDA software, generates input parameters and files automatically, and offers user-friendly visualization of output results. webSDA is useful for beginners learning how to perform Brownian dynamics simulations as well as for experts preparing input files for running simulations. Simulation times and file sizes are limited on the webSDA server so webSDA allows the generated input files to be downloaded for carrying out production runs with SDA on local or high performance computing resources. We believe that webSDA will be an easy-to-use tool for users who are interested in using Brownian dynamics simulations to study biological systems. Currently, webSDA does not support all the functions of SDA. However, webSDA has a modular structure that will facilitate the implementation of further methods and features in the future.
